# Advances in single-cell transcriptomics in animal research

**DOI:** 10.1186/s40104-024-01063-y

**Published:** 2024-08-02

**Authors:** Yunan Yan, Senlin Zhu, Minghui Jia, Xinyi Chen, Wenlingli Qi, Fengfei Gu, Teresa G. Valencak, Jian-Xin Liu, Hui-Zeng Sun

**Affiliations:** 1 Institute of Dairy Science, Ministry of Education Key Laboratory of Molecular Animal Nutrition, College of Animal Sciences, Zhejiang University, Hangzhou, 310058 China; 2 Key Laboratory of Dairy Cow Genetic Improvement and Milk Quality Research of Zhejiang Province, Zhejiang University, Hangzhou, 310058 China; 3 Agency for Health and Food Safety Austria, 1220 Vienna, Austria

**Keywords:** Disease modelling, Genetics, Health, Livestock, Nutrition, Reproductive performance, Single-cell RNA sequencing

## Abstract

Understanding biological mechanisms is fundamental for improving animal production and health to meet the growing demand for high-quality protein. As an emerging biotechnology, single-cell transcriptomics has been gradually applied in diverse aspects of animal research, offering an effective method to study the gene expression of high-throughput single cells of different tissues/organs in animals. In an unprecedented manner, researchers have identified cell types/subtypes and their marker genes, inferred cellular fate trajectories, and revealed cell‒cell interactions in animals using single-cell transcriptomics. In this paper, we introduce the development of single-cell technology and review the processes, advancements, and applications of single-cell transcriptomics in animal research. We summarize recent efforts using single-cell transcriptomics to obtain a more profound understanding of animal nutrition and health, reproductive performance, genetics, and disease models in different livestock species. Moreover, the practical experience accumulated based on a large number of cases is highlighted to provide a reference for determining key factors (e.g., sample size, cell clustering, and cell type annotation) in single-cell transcriptomics analysis. We also discuss the limitations and outlook of single-cell transcriptomics in the current stage. This paper describes the comprehensive progress of single-cell transcriptomics in animal research, offering novel insights and sustainable advancements in agricultural productivity and animal health.

**Supplementary Information:**

The online version contains supplementary material available at 10.1186/s40104-024-01063-y.

## Introduction

Cells, the basic unit of life, vary widely in shape, size, and gene expression. It is vital to explore the different biological properties of individual cells in complex tissues to understand the process of life activities. The invention of flow cytometry at the end of the 1960s was a major breakthrough in both qualitative and quantitative measurements of cellular characteristics, as well as cell sorting [1]. It remains a widely applied strategy for single-cell analysis and isolation to date. In 1990, polymerase chain reaction was introduced to amplify DNA or RNA in individual cells, providing even more functional information [2], which further demonstrated that transcriptomic studies on individual cells were feasible. However, this method is limited by amplification bias and low throughput. The development of first-generation sequencing techniques made significant progress in molecular sequencing technology, but the costs remained high, and the sequencing throughput was low [3, 4]. Next-generation sequencing technologies, such as “sequencing by synthesis” and accelerated parallel sequencing, have successfully overcome the above limitations, and RNA sequencing (RNA-Seq) is the best-known and most commonly used approach to date [5]. RNA-Seq involves total RNA extracted from organs, tissues, or a group of cells, obtaining average transcriptomic data while often masking specific information of individual cells in the population. To systematically study complex biological processes at higher resolution and reveal functional heterogeneity within tissues, researchers have developed a series of technologies, including multidimensional studies at the single-cell level. In 2009, Tang’s team developed the single-cell RNA sequencing (scRNA-Seq) method, enabling large-scale access to gene expression information from individual cells [6]. Since then, the use of scRNA-Seq has undergone rapid growth and evolution. In 2018, the journal “Science” ranked scRNA-Seq at the top of its list of the year’s most noteworthy technologies. Using scRNA-seq, international research projects, such as the Human Cell Atlas (https://www.humancellatlas.org/), have been launched to identify all cell types involved in human development, health, and disease. Notably, a collaborative project consortium focusing on livestock, FarmGTEx (http://farmgtex.org/), joined forces in the single-cell transcriptomic research of different livestock species by bringing together researchers from around the world to discover regulatory variants, molecular targets, and phenotype predictions at the single-cell level. Single-cell technologies are now emerging at multiple molecular levels, including genomics, transcriptomics, epigenomics, proteomics, and metabolomics, at continuously enhanced resolution, accuracy, and efficiency, setting the stage for the practical application of single-cell technologies.

**Fig. 1:**
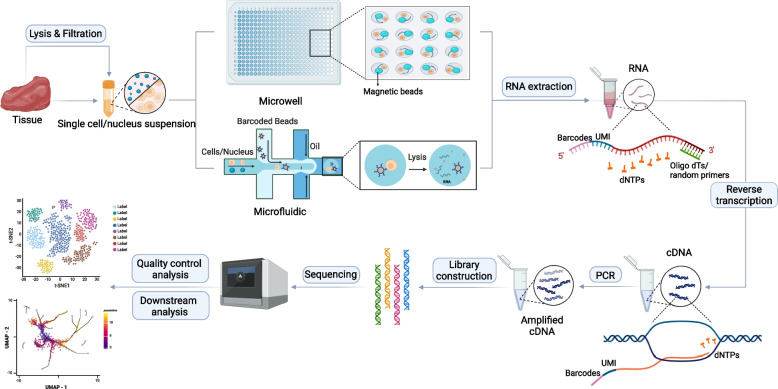
The workflow of single-cell/nucleus RNA sequencing

Single-cell RNA sequencing is a technology that can be used to comprehensively reveal the gene expression profiles of cells by sequencing transcripts in individual cells one by one [7]. It is currently the most mature technology for high-throughput functional resolution at the single-cell level. As shown in Fig. 1, the process begins with the dissociation of fresh tissue into a single-cell suspension, followed by the selection of different single-cell capture strategies depending on the cell numbers. Manual operations (e.g., limited dilution, laser cutting, micromanipulation) are generally chosen for small cell numbers, but microfluidic and microwell techniques are used when the cell throughput is on the order of tens of thousands [7]. After single-cell capture, cDNA is obtained through reverse transcription, amplified, and then sequenced. Downstream analysis can be conducted after completion of the single-cell sequencing run. The analysis process typically involves 3 stages: primary analysis (base detection), secondary analysis (multiple isolation, alignment, and genetic identification), and tertiary analysis (data visualization and interpretation) [8]. As scRNA-Seq involves a sufficient number of active cells from fresh samples, it is impossible to perform it efficiently from frozen or indigestible samples. The emergence of modern single-nucleus RNA sequencing has solved this problem by inserting an extraction procedure to extract the nuclei of single cells before isolating and labelling the nuclei, making it possible to detect nuclear gene expression at the single-cell level [9, 10]. However, this method is currently not applicable for immune cells, and sequencing sensitivity may be low due to the low abundance of mRNA in the nuclei of some cells. Overall, single-cell transcriptomics has allowed us to fully decipher cell types/subtypes and functions in almost all species by constructing a reference catalogue of gene expression encompassing cells throughout the body. In addition, single-cell transcriptomic data can be visualized in multiple dimensions via bioinformatics analyses, such as differential enrichment and proposed time-series analyses. These strategies enable the reconstruction and simulation of biological system operations.

Livestock produces more than 15% of the world’s high-quality protein. The Food and Agriculture Organization of the United Nations predicts that by 2050, up to 50% of animal food will be required to feed 10 billion people. Rapidly emerging bioscience biotechnologies have assisted animal science experts in producing even more animal products with limited resources. However, when facing new challenges, such as studying the biological factors determining economic traits, product quality, and animal health, it is crucial to use high-throughput methods, especially single-cell transcriptomics, to better investigate cellular functions and interactions. Relevant research has already been conducted in animal studies worldwide using scRNA-Seq (see Additional file 1 for details). For example, identifying and annotating various cell types in livestock can reveal their specific functions and interactions, which is crucial for obtaining an even better understanding of the cellular composition, differentiation status, and intercellular regulatory networks in animal tissues and organs. This technology provides high-resolution information on gene expression in individual cells, facilitating the discovery and comparison of expression differences between cells and allowing for the identification of key genes related to economically important characteristics, nutrient metabolism, and disease resistance. In addition, scRNA-Seq can enable the discovery of new genes and regulatory elements, particularly those that are poorly expressed or cell-specific. Thus, our understanding of livestock animal genomes is enhanced, and a new approach to functional studies is provided. The transcriptomic changes in cells can be tracked at different time points via scRNA-Seq, revealing the dynamic processes of cell development, differentiation, and functional transformation. This information is important for understanding tissue growth, development, regeneration, and immune responses in livestock.

Our paper provides an overview of the use of scRNA-Seq in livestock research in recent years. It also offers insights and references on the single-cell data analysis process, which can serve to make scRNA-Seq a more robust tool for research on animal husbandry and breed optimization.

## Applications of single-cell transcriptomics for livestock husbandry

### Exploration of nutrient metabolism and immune responses

**Fig. 2:**
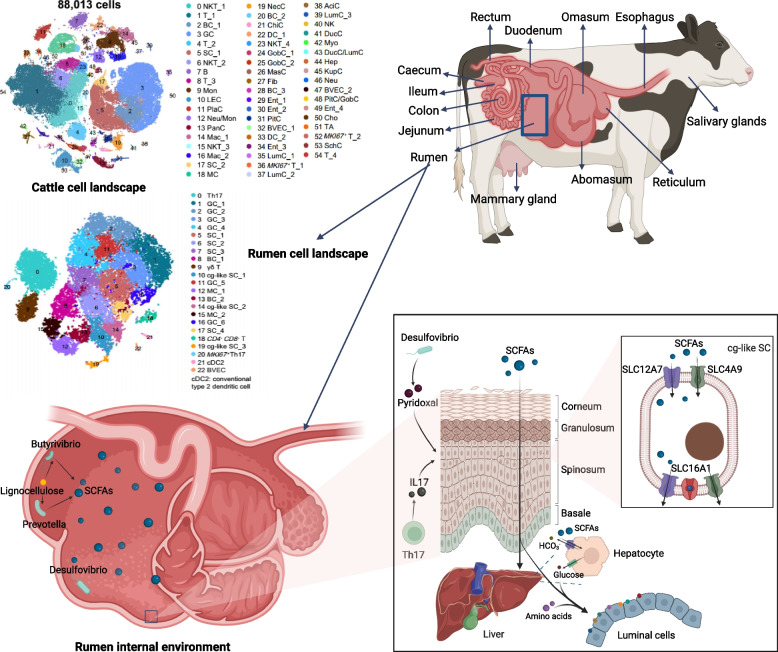
Functional mechanism of rumen short-chain fatty acids uptake

The gastrointestinal tract (GIT) serves as a vital organ for nutrient absorption, and the mucosal immune system in animals largely affects production performance, animal welfare, and the safety of livestock products. Through single-cell sequencing of the livestock GIT, we will be able to investigate the composition and distinct metabolic patterns of various cell types, and ultimately, we may provide precise targets for nutritional manipulation and enhance nutrient absorption in these animals. Among the GIT in ruminants, the rumen holds undeniable significance for nutrient digestion and absorption, particularly for short-chain fatty acids (SCFAs), which are absorbed by the rumen epithelium and can meet 70%–80% of the body’s energy requirements [11]. However, the rumen epithelium is a complex structure consisting of four layers of cells of different types and functions. This complexity has hindered mechanistic explorations of rumen epithelial absorption and turnover of specific nutrients. Earlier scRNA-Seq work has shown that spiny cells of the rumen epithelium play a crucial role in SCFAs [12], but more detailed cell subtypes were not identified due to factors such as the lack of identified marker genes. The application of the newly developed rumen single-cell suspension preparation method for scRNA-Seq has given rise to even more comprehensive insights into rumen epithelial cell types. Wu et al. [13] integrated and expanded specific marker genes for bovine cell lineages based on previous studies; 20,728 rumen epithelial cells were clustered into 18 rumen epithelial cell types, and the specific metabolic characteristics of each cell subtype were characterized. The ability of different epithelial cells to absorb SCFAs was explored by analysing the expression of genes encoding transporter proteins and scoring the related functional pathways. The findings revealed that channel-gap-like (Cg-like) spinous cell regulated by IL-17 was the preferred subtype for SCFA absorption in dairy cows [13]. The microbiota is essential for nutrient absorption in the GIT, and further joint mining of rumen metagenomic data characterized the interactions between Cg-like cells and fibre-degrading bacteria via structural domains of the secreted proteins (Fig. 2) [14]. Unfortunately, the study did not address ruminal nutrient uptake during different developmental stages. Studies on intestinal nutrient absorption in monogastric animals based on single-cell transcriptomics have primarily concentrated on cross-species comparisons of distinct cell types and functional analyses. Specifically, studies have examined significant interspecies differences and regional characteristics in hormone-secreting enteroendocrine cell [15, 16]. For example, there is a gradual decrease in the number of these cells after the birth of piglets [17].

The immune system is vital for animal health and plays a pivotal role in defending against external hazards by maintaining physiological equilibrium. The application of scRNA-Seq for investigating livestock immunity has generated valuable insights. The advent of scRNA-Seq has contributed to an in-depth understanding of functional heterogeneity, molecular regulatory mechanisms, and infection responses among cellular subsets within central immune organs (e.g., the bursa and thymus) and peripheral immune organs (e.g., Pyle’s collecting lymph node) [18–20]. However, investigations on the bone marrow of domestic animals remain a major issue. Single-cell transcriptional profiling of various immune cells, such as peripheral blood monocytes and lymphocytes of domestic animals, underlines the homology and species specificity of immune gene expression, providing a molecular basis for understanding the immune response process in livestock [21–31]. For example, single-cell transcriptomic data of peripheral blood mononuclear cells (PBMCs) following disease in domestic animals have been used to identify pathogenic immune cell subpopulations and transcriptional modules driving pathogenesis [32]. This approach has helped with identifying subpopulations and molecular markers of PBMCs in chickens infected with avian influenza and avian leukemia virus [33–35], unravelling key signalling factors associated with lipopolysaccharide-induced glycolipid metabolism abnormalities in cattle [22], and uncovering cell types and genes linked to periparturient immunosuppression in dairy cows [36]. In recent years, single-cell sequencing research has shifted towards porcine immune cells during piglet weaning stress, weaning diarrhoea, and intestinal inflammation, as it enables the tracking of immune cell heterogeneity during pathogenic microbial infections and host responses during diseases. For example, scRNA-Seq analysis revealed that tumor necrosis factor-α secreted by different immune cells could contribute to disease, as observed in studies investigating African swine fever and porcine intestinal inflammation [37, 38]. These observations have opened new avenues for the development of novel vaccines and targeted therapies. In summary, scRNA-Seq studies allow for the precise identification and analysis of cell types and their interactions involved in the immune processes of domestic animals. They also allow for the examination of regulated genes, signalling pathways, transcription factors, and immune cell subpopulations with potential pathogenic functions. Current studies primarily focus on exploring immune mechanisms, however the potential of scRNA-Seq could be harnessed to evaluate the efficacy and response to immunotherapy. This involves concentrating on T cells, which are important players in the immune response and play key roles in animal health.

### Investigation of cell factors for animal reproductive performance

The level of reproductive performance in livestock, which can directly affect productivity, depends on the ability to produce high-quality male and female gametes. The use of scRNA-Seq combined with proposed time-series analysis has allowed for the careful investigation of the dynamic mapping of cell fate transitions and gene expression changes during spermatogenesis in dairy goats, sheep, yaks, and premature piglets [39–45]. Through comprehensive analyses, researchers have identified specific marker genes and key signalling pathways related to germ cells within the testes of farm animals. Furthermore, studies have explored the homology and differences in male germ lines across different species, expanding our understanding of testicular development and spermatogenesis [40, 46, 47]. It has become evident that spermatogenesis relies on an ecological niche composed of testicular somatic cells. Consequently, studies have increasingly focused even more on single-cell sequencing analyses of these cells over the past few decades. However, due to variations in study samples and resolution, there are significant knowledge gaps related to the classification of testicular somatic cells, necessitating further investigation [40, 42–44, 46]. The growth and development of ovarian follicles are primarily regulated by granulosa cells. Through single-cell transcriptomics, the heterogeneity and differentiation pathways of follicular cells and granulosa cells in the ovaries of livestock animals have been characterized [48–52]. However, intercellular interactions within the ovary remain poorly understood. Recent studies in yaks, goats, and domesticated pigs have attempted to fill this gap [53–56]. For instance, Chen et al. discovered that porcine ovarian mural granulosa cells primarily engage in intercellular communication with cells of the same type, whereas ovarian theca granulosa cells predominantly emit signalling cues to different cell types [53]. Nevertheless, these studies are based on relatively small sample sizes, and more in-depth work is needed to corroborate their findings. High-precision mapping of germ cell genesis in livestock animals has enabled researchers to conduct in-depth studies on the screening, diagnosis, and treatment of reproduction-related disorders, such as sperm damage, abnormal oocyte development, and male infertility, in the progeny of interspecifically crossbred individuals [57–59]. The regulatory characteristics and interspecific differences of various types of cells in the gonads of livestock animals obtained at specific time points represent another research focus that can be assessed by single-cell transcriptomics [60–62]. To date, continuous developmental differentiation in livestock gonads is poorly understood. Only the gonadal differentiation of the chicken embryo has been analysed, and the discovery that the supporting cells during gonadal differentiation in the chicken embryo are derived from mesenchymal stem cells, in contrast to other vertebrates, has revolutionized our previous understanding of gonadal cell types [63]. Notably, noncoding RNAs significantly affect the regulation of germ cell proliferation and differentiation in livestock [40, 64]. However, capturing these noncoding RNAs remains challenging, necessitating advancements in the application of this technology to enhance our understanding of livestock reproductive performance [65].

Single-cell transcriptomics has also been widely accepted as an efficient tool for investigating cell fate and transcriptional regulation during embryonic development. Studies employing scRNA-Seq have examined embryos at various life stages in livestock, such as cattle, pigs, chickens, sheep, and rabbits, thus making dynamic transcriptional profiles and cellular differentiation trajectories accessible within each germ layer of early-stage embryos [66–74]. For example, these analyses have uncovered species-specific features of early embryonic development (E5-E13) in pigs, revealing the differentiation of bovine trophoblast mononuclear cells into binucleated cells and the changes in differential gene expression and associated signalling pathways from the 8-cell to the mulberry embryo stage in sheep [67, 68, 71]. By leveraging scRNA-Seq, researchers can also analyse embryos with developmental abnormalities or those transferred from in vitro cultures. These investigations contribute to identifying potential causes of developmental abnormalities, offering valuable guidance for improving reproductive techniques such as in vitro fertilization and embryo transfer. It has been observed that incomplete activation of certain metabolic pathways leads to metabolic abnormalities. Epigenetic modification may be responsible for the significant effects on subsequent pregnancy and calving rates in females, and comparing the developmental transcriptional profiles of embryos transferred in vitro from different states [75] may provide new ideas for the treatment of embryonic developmental abnormalities.

### Elucidation of genetics and developmental biology in livestock

Throughout the ontogeny of an organism, the transcriptome of certain cells undergoes substantial transformation. scRNA-Seq represents a novel approach for elucidating the dynamic patterns of gene expression during livestock genetic development and for revealing the regulatory networks governing developmental and evolutionary processes. The ultimate goal is to depict the trajectory of cellular fate transformations. Developmental mapping has been performed on the nervous systems and skeletal limbs of monogastric animals [74, 76–78] while focusing on the nodes and key cell types involved in the developmental differentiation of organs or tissues. It is worth mentioning that aided by single-cell transcriptomics of embryonic limbs in poultry, studies have pinpointed pivotal regulators and signalling pathways driving limb differentiation formation along with their regionalization for the first time [79, 80]. Among ruminants, rumen development has received significant attention. Scientists have constructed a comprehensive developmental landscape of thirteen metabolic tissues in ruminants [81, 82], which encompasses a holistic comparison of heterogeneity within rumen epithelial cell types, cell functions and interacting microbiota between calves and adults. These findings suggested that calf epithelial progenitor cells exhibit greater differentiation potential and display greater activity in cell proliferation, differentiation, and innate immune responses [81, 82]. Conversely, adult bovine cells show prominence in immune cells and have increased activity during antioxidant, adaptive immune, and fatty acid metabolism processes [81, 82]. Similarly, studies have shown that the keratinization process during rumen epithelial cell development is associated with the cessation of keratinocyte differentiation at specific stages [12, 83]. Previous studies have successfully identified key genes associated with rumen growth and development by scRNA-Seq of the rumen epithelium [84, 85]. Overall, scRNA-Seq is assisting in the construction of a developmental evolutionary tree of livestock from the embryo to the mature individual. In single-cell transcriptional studies on animal genetic development, previously unidentified cell types are discovered, such as a specific class of endothelial cell clusters found in yak lungs, were revealed as potential factors in plateau acclimatization, and it was shown that calf-specific STOML3^+^ cells have the potential to maintain the internal environment of the liver [82, 86]. In addition, the exploration of unexpected functionalities harboured by common cell types has become possible, e.g., luminal epithelial cells could be involved in both lactation and immune responses [13]. It is worth mentioning that whole-body single-cell atlases have been generated for several mammalian species [87–91], with those in humans and mice covering the entirety of life stages [92]. However, to date, comprehensive whole-body single-cell atlases are still lacking for livestock animals.

The comparison of single-cell transcriptomic data from high- and low-productivity livestock has become an approach to identify genes and key cell types related to economic traits and has led to the selection and breeding of highly productive livestock breeds. Taking meat production traits as an example, the quantity and quality of meat production largely depend on the development of skeletal muscle. Analyses of the single-cell transcriptomics of skeletal muscle from fat and lean pigs could effectively identify key genes for adipocyte differentiation and epigenetic modifications [93–95]. By analysing the developmental trajectories of myogenic progenitor cells, it was observed that the skeletal muscles of lean livestock were more closely related to myogenic progenitor cells and more responsible for muscle development than the skeletal muscles of fat pigs, suggesting that mechanistic explorations of myogenesis can lead to the study of differences in genealogical cell differentiation [93, 96]. The identification of specific liver cell clusters between laying and non-egg-laying populations in egg-laying birds may also provide new insights for improving egg production in the future [97]. More information can also be acquired from single-cell transcriptomic studies of niche economic traits such as fleece- and silk-producing traits. The former is limited by the asynchronous development of the wool bursa [98], and the latter is limited by the understudied organ of the silk gland; neither the wool bursa nor silk glands have been accurately analysed previously for specific cell types. The emergence of scRNA-Seq has enabled in-depth knowledge of the distribution of the composition of each cell type and the trajectories of key cells during the different developmental periods of the wool bursa and silk glands [99–102]. Additionally, new marker genes involved in the synthesis of velvet and silk proteins have been identified [100], which can improve the quality of cashmere and silk. For instance, the *ACTA2*, *COL1A1*, and *CLCL6* genes may regulate cashmere fineness [103]. Although single-cell transcriptomic analyses have shown promise for improving genetic breeding, current research results are difficult to apply in animal production or have limited impact. Future research concentrating on diseases that are commonly associated with production should follow. For example, particular cell subpopulations associated with mastitis in dairy cows can be identified.

### Emergence of disease models

The utilization of scRNA-Seq in model animals is highly important, especially in pigs, an animal commonly used as a medical/disease model [104–106]. By employing scRNA-Seq, researchers have gained profound insights into the composition of organelle types between pigs and men, thereby revealing the heterogeneity and conservatism of gene expression and regulatory mechanisms within the biological processes of interest and promoting in-depth biomedical research and widespread use of the domestic pig as an animal model. Previous studies have focused on individual tissues or organs (e.g., immune cells [15, 18, 23, 24, 27, 60], lungs [107], liver, embryos [108], the reproductive system [46, 47, 109], and the digestive tract [16, 110]) in pigs. These studies utilized cross-species analyses to identify conserved or specific gene modules in tissues or screened for cellular subpopulations and risk genes associated with disease. Moreover, they have revealed gene–trait associations through modelling or induction. Pig lung tissue data were characterized for both similarities and differences in cellular communication and expression patterns of respiratory virus receptors in each cell type of the lung compared with human lung tissue data [107]. A comprehensive porcine brain atlas has facilitated the identification of cell types and risk genes linked to eight neurological disorders, e.g., attention deficiency [111]. Data from the pancreas have suggested that *TXNIP*, a stress gene in acinar cells, could become a potential target for the treatment of diabetes [112]. The investigation of the gallbladder in neonatal piglets has shed light on the mechanisms of cystic fibrosis-related hepatobiliary disease [113]. For the first time, a pioneering study constructed a porcine single-cell atlas database that comprehensively describes the heterogeneity of cells among 20 tissues/organs in pigs [114], providing a global view of tissue differences between domestic pigs and humans. Rather than focusing on specific cell clusters, Wang et al. [114] emphasized distinct functions and typical markers of endothelial cells commonly involved in different tissues and suggested that the endothelium may interact with cells through the VEGF, PDGF, TGF-β, and BMP pathways. Microglia have also been noted to be highly conserved across species during evolution [114], strongly supporting the view of pigs as an invaluable data resource for research on human diseases. In the context of xenotransplantation, pigs are considered the most suitable donors for human organ transplantation. However, the occurrence of rejection has restricted the application of this technique [115]. The expression patterns of ten genes associated with human immunobiological incompatibility and dysregulation of coagulation have been obtained across different cell types in pigs [107]. This discovery holds the potential to enhance the immunocompatibility of porcine xenotransplantation in the future through targeted genetic engineering, thereby improving survival after organ transplantation [116]. However, is important to note that the current study does not adequately address the influence of physiological states and manipulation on the samples. These factors may affect the results by leading to differing numbers of captured cells and altered cell typing. Future research should address this crucial aspect.

In addition to pigs, chickens and rabbits serve as valuable disease models for constructing single-cell reference maps. Differential cellular components can be identified by comparing “disease maps” with “normal maps”, which may in turn predict molecular disease mechanisms. For example, rabbits have been used to study mammalian cardiac contraction, proto-gut embryonic development, proliferative vitreoretinopathy, and hyperlipidaemia-induced spongiosis [72, 117–119]. Similarly, chickens have proven valuable for investigating retinal development, hearing damage, and the mechanism underlying melatonin-related weight loss [120–122]. Among wild animals, antlers, a unique mammalian appendage capable of complete natural regeneration, have been demonstrated to grow similarly to long bones in humans. Leveraging the potential of scRNA-Seq, scientists have revealed key cell types and differentiation trajectories involved in antler regeneration [123–125], opening new avenues for exploring mammalian organ regeneration and organ damage repair. Future research should address the current limitations in genome annotations for reindeer [123–125].

## Experience in single-cell transcriptomic data analysis

### The optimal sample size for single-cell transcriptomic research

Increases in sample size and sequencing depth could result in the discovery of new and rare cell types, encompassing both previously unrecognized entities and those present in tissues where they have not been detected before [126]. For instance, among 42,182 cells from three forestomach samples, T helper 17 and epithelial stem and progenitor cells were first identified in dairy cows [13]. A total of 29,231 individual cells were obtained from three samples of porcine adipose tissue, from which subtypes of cells transformed from endothelial to mesenchymal cells were distinguished [114]. Compared to studies in livestock, there are more instances of new cell type identification in humans and model organisms, such as a new specialized uroepithelial cell type discovered among 25,307 cells across three bladder samples in humans and mice [127] and four clusters of nonsensory epithelial cells of the ampulla identified from four stages of mouse crista ampulla samples [128]. Based on the aforementioned examples of novel cell discoveries, it is evident that employing three or more biological replicates and analysing over 20,000 cells significantly enhances the likelihood of uncovering new cell types. Considering the spatial positioning of tissue sampling and individual differences in cell dissociation, a larger number of biological samples further provides advantages in elucidating the comprehensive distribution of cell types. However, due to the high cost of reagents and sequencing (a total of 10–15 K RMB per sample), excessive sampling may cause more significant input and divert focus from validation experiments. As an illustration, in cell atlas studies, larger biological sample numbers are preferable for identifying cell types at higher resolution [21, 44, 102]. However, if the focus is solely on identifying which cell type is responsive to an experimental treatment, one biological replicate may be effective, as that one cell type typically consists of more than 20 cells/transcriptomes [129, 130]. In addition, time-series designs generally involve fewer biological replicates than two-group comparisons [36, 129].

With the development of single-cell sequencing technologies, an eight-channel microfluidic system that can capture up to 10,000 cells per channel has been developed, allowing for simultaneous detection of sample cell counts ranging from 50,000 to 800,000 cells in a single run [131], making a single sample cover a large number of cell/transcriptomic replicates. Bioinformatics tools allow us to enhance and refine the power of single-cell data analysis through the interpretation of transcriptome data. Deconvolution algorithms are now being employed to dissect bulk transcriptomic data to the single-cell level [132], increasing the statistical power of bulk transcriptomics in single-cell experiments. In addition to deconvolution algorithms, a framework for integrating single-cell RNA sequencing, epigenomic SNP-to-gene maps and genome-wide data enables the identification of target cell types based on the strong statistical power of GWAS data. Currently, emerging large-scale single-cell pre-trained models with tens of millions of cells, such as scGPT, can empower the tasks of our small datasets, including cell classification, network inference, and transcription factor perturbation analysis [133]. Taken together, researchers need to determine the amount of biological replication that is sufficient to capture biological variability and provide statistically significant results while considering the cost of experiments and the complexity of data analysis. Bulk sequence data and large-scale pre-trained models could be used to enhance the statistical power of single cells.

### The number of cell clusters that optimally match the real situation

**Fig. 3:**
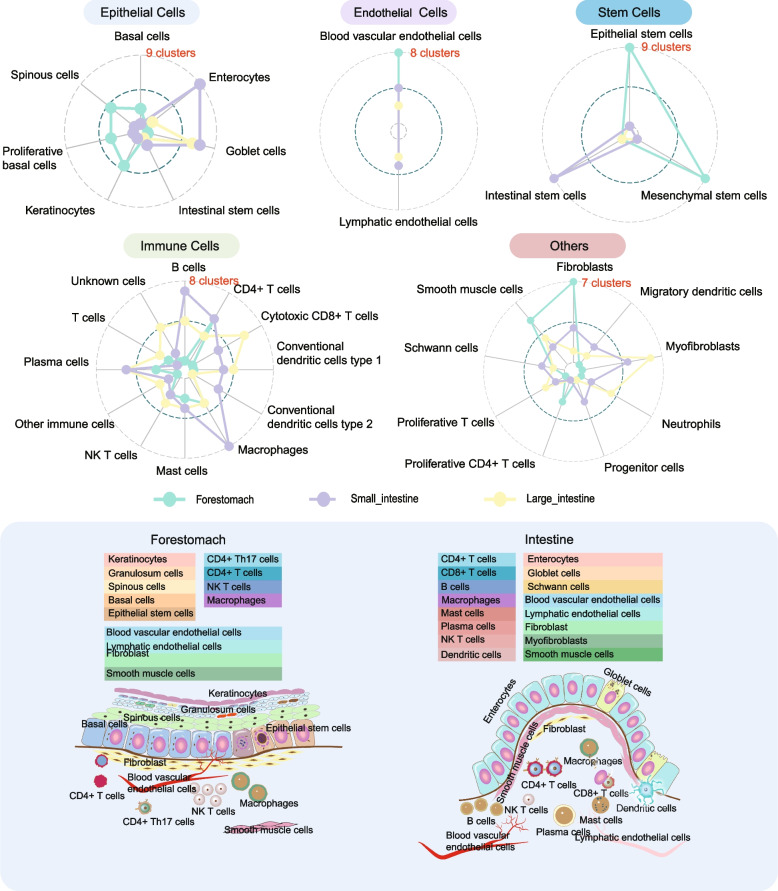
A summary of the common cell types identified in the gastrointestinal tract of livestock

In brief, the number of cell types is determined by clustering algorithms; specifically, after obtaining single-cell datasets, the cells can be categorized into 2–5 major clusters based on automatic annotation and positional variance in the dimensionality reduction results. With major biological classifications, multiple higher resolutions could be used to identify the more specific cell types corresponding to the basic biological knowledge of a given tissue (Fig. 3). As an example, the large intestine comprises diverse epithelial cell types arranged in distinct configurations. Absorptive enterocytes predominate, lining the villi and crypts and specializing in the uptake of water, electrolytes, and nutrients from the luminal contents [134]. Goblet cells interspersed throughout secrete protective mucus, creating a mucosal barrier against pathogens and toxins [135]. Enteroendocrine cells release hormones to regulate digestive functions, while Paneth cells, predominantly found in the small intestine but also present in smaller numbers in the large intestine, contribute to innate immunity through the secretion of antimicrobial peptides [136, 137]. Mesenchymal cells, immune cells, endothelial cells and neural cells are present in the large intestine. However, the epithelium of the intestine is usually composed of a single layer of columnar epithelial cells, such as those with 1 or 2 clusters, and some cuboidal cells, such as goblet cells, with 2 or 3 clusters [21]. Different resolution values should be tested to provide the finest demonstration of the known cell types within each tissue. Furthermore, based on the objectives and experimental design, the resolution and number of cell clusters may vary while maintaining biological features. A greater number of clusters is essential for key subcluster identification. For instance, specific macrophage and conventional dendritic cell subsets were identified as key mediators of cellular cross-talk in the colon tumour microenvironment from 54,285 cells divided into 40 clusters [138].

Moreover, clustering algorithms should also be essential for generating the number of cell clusters. First, suitable algorithms should be chosen based on the cell numbers. The K-nearest neighbours algorithm is typically applied to datasets with small cell numbers, with the number of clusters predetermined before clustering. Moreover, the Louvain and Leiden algorithms are normally used for single-cell datasets with more than 100,000 cells, with the number of clusters determined by the resolution set by the analyser [139, 140]. The number of clusters can be determined by intra- and intercluster similarity, community detection, eigenvector-based metrics, and stability [141]. However, these four predominant clustering methods lack systematic evaluation strategies for estimating the number of cell types. Therefore, stability-based approaches for estimating the number of cell types, such as scCCESS, have been proposed [141], enabling the estimation of the number of cell clusters without the need for researcher observation. Some bioinformatic visualization tools also help us estimate the number of clusters. The Clustree algorithm [142] employs a bottom-up hierarchical clustering approach for cluster information at different resolutions, which facilitates our selection of a suitable resolution by incorporating biological insights.

### A well-formulated strategy for cell type identification

The identification of cell types is a fundamental and essential step in the analysis of single-cell transcriptome data and is accompanied by dimension reduction and clustering [143]. Generally, the number of cell types and the annotation results should be consistent with the corresponding physiological characteristics.

The common cell types identified in the GIT of livestock are summarized in Fig. 3. The clusters can be classified into five main cell groups: epithelial cells, endothelial cells, stem cells, immune cells and others. We noticed that the number of clusters for immune cell types in the forestomach is generally lower than that in the intestine, while fibroblasts and muscle cells were more diverse. In the intestine, specifically in the small intestine, B cells and macrophages are more abundant. The cluster numbers correspond to substantial peristalsis and mechanical wear in the foregut, as well as the clear ability of the small intestine to eliminate exogenous invading microorganisms [144]. Collectively, these cells form a complex biological system responsible for digestion, nutrient absorption, protection against pathogens, and tissue repair and regeneration. Based on the previous cell group summary, we propose three approaches that are beneficial for identifying cell types: (1) collecting biological background information in a given situation; (2) referencing standard cell type names and databases; and (3) assisting in cell type identification with the help of bioinformatic software. It is necessary to summarize basic knowledge in the process of annotating cell types, including biological knowledge and standards/universal naming rules. Regarding how many types of cells should be classified, an effective method is to summarize the physiological characteristics of animal tissues through the accumulation of relevant experimental data and scientific research results [143]. For example, according to basic knowledge, the cell types present in the epithelial tissue of the rumen of dairy cows consist of four layers of epithelial cells (basal, spiny, granular, and stratum corneum; stratum corneum cells are dead cells and will not be captured), immune cells residing in the tissue, endothelial cells in the connective tissue, smooth muscle cells, and fibroblasts [13]. Therefore, the presence of these cell types can be used as a reference when determining whether the number of cell types is accurate and whether the current clustering parameters should be adjusted. In animal science research, genes related to animal production or health traits may have corresponding biological significance to cell type. For example, Wu et al. [13] found a new subcluster of rumen epithelial cells called channel gap-like spinous cells in dairy cows via an in-depth analysis of solute carrier gene expression. Additionally, Wang et al. identified EndMT cells as critical for endothelial-to-mesenchymal transition (EndMT) based on gene expression in stromal and endothelial cells [114].

To make the cell type identification results more biologically meaningful, the integration of public databases and bioinformatics analysis software should be considered. Public databases of single-cell transcriptome data, such as the Human Cell Atlas, incorporate marker genes for cell type identification and standard or universal cell type nomenclature rules [145]. In animal science research, the cattle cell atlas constructed by Wu et al. [82] and the pig cell atlas constructed by Wang et al. [114] are also of high reference value, although they do not cover all tissue types. A unified system will improve efficiency and applicability for other studies [146]. Clustree software can be used for determining whether the number of cell types obtained from clustering is appropriate [142]. Software that automatically annotates cell types can also assist in identifying cell types, e.g., Cellhint [146], CellTypist [147], and SingleR [148]. Software programs for the automated annotation of cell types have been developed based on mathematical models and are specific for annotating certain cell types. For example, Cellhint is used to annotate human cells, and CellTypist is only used to annotate human immune cells. Notably, bioinformatics analysis should be performed manually to ensure the biological significance of the annotated results.

## Outlook

Currently, single-cell transcriptome sequencing technology is cumbersome and costly in terms of experimental steps. Professional experimentation and specialized software support are required for all aspects, from sample preparation to data analysis. Moreover, the bias of cell capture during the sequencing process may lead to inaccurate results, which is mainly attributed to the possibility of missing or detecting specific types of cells at low frequencies. Moreover, bioinformatics tools and algorithms are still suboptimal, making it challenging to extract meaningful information from the massive amount of data. In the future, it will be necessary to simplify and optimize the process of single-cell technology to reduce its cost and operational difficulty and to make it easier to apply in different fields. The accuracy and efficiency of cell capture techniques should be improved to reflect the diversity of cellular communities. In the meantime, techniques for cell capture and mRNA enrichment in prokaryotes are still under development. The implementation of microbiome single-cell transcriptome technology will substantially broaden the application landscape of single-cell technology [149].

One of the greatest limitations of scRNA-Seq is the loss of spatial information due to tissue dissociation. Spatial transcriptomics is a more recently developed methodology that allows for the localization and construction of a cellular expression map with a spatial dimension, which is not achievable with scRNA-Seq. Coupled with scRNA-Seq, this approach can stereoscopically demonstrate the heterogeneous distribution and functional localization of individual cells and reveal spatial differences in cells during evolutionary development or disease onset. However, the application of this approach to domestic animals is still relatively rare. Only studies on the spatiotemporal transcriptional profiling of chicken heart development have been reported, but have revealed the pathways through which cardiac cell differentiation and morphological changes occur at the same time as spatially restricted regulatory programmes [150]. The complexity of life activities is difficult to determine by single-modality omics methods; therefore, single-cell multi-omics technologies, including single-cell transcriptome as the core analysis combined with genome, proteome and metabolome analyses, are inevitable [151] and will be able to deepen the understanding of cell type and state in greater dimension. For example, the dynamic changes of single-cell RNA/ATAC sequencing in porcine embryonic skeletal muscle were consistent with the activity of different cell type-specific transcription factors, which helped identify key regulators of muscle formation after integrative analysis [152].

Sequencing of certain animals or organs can be challenging due to their limited availability, difficulty in manipulation, or ethical concerns. Organoids, 3D cultures developed from stem cells that closely resemble the source tissue [153], are valuable in vitro tools for organoid single-cell transcriptomics in such cases. Integrating organoids with scRNA-Seq can allow more precise comparisons between organoids and source tissues in terms of cell types and gene expression patterns; moreover, through real-time monitoring of transcriptome changes in single cells of organoids cultured under different treatments, it is possible to provide potential cellular and molecular phenotypic information on complex traits, such as feed efficiency and disease resistance. Organoid models have been constructed for various livestock species [154–156] and are mainly used to study organ development, host–microbe interactions, cellular nutrient metabolism mechanisms, and drug toxicity. Zhang et al. [83] utilized a significant quantity of butyric acid to stimulate rumen organoids, and the organoids showed noticeable keratinization and significant increases in the expression levels of related keratinization genes. These findings were confirmed by single-cell sequencing, demonstrating the potential for combining single-cell transcriptomes and organoids for the study of biological mechanisms.

Single-cell transcriptomics reveals the role of different cells in organisms. However, it is important to recognize that in multicellular organisms, the functionality of the organism is dependent on synergistic interactions between different cells. Thus, single-cell transcriptome-based research should consider the relationships between cell lineages and the interactions between cells. This might also prevent an overemphasis on the functions of individual cells and might rather ensure that the influence of the hostile environment on cell development and function is not ignored. Using a multidimensional approach to single-cell transcriptome studies, such as cell communication analysis, the interaction network between cells can be reconstructed and predicted, revealing signalling and regulatory mechanisms and providing a more accurate reference for studying overall organism and tissue function. It is also possible to validate the accuracy of single-cell sequencing data through various biological experimental means, ensuring a more reliable assessment of research results.

## Conclusions

Single-cell transcriptomics has enabled the analysis of heterogeneity in gene expression in tissues and organs at the single-cell level. This advancement has provided a robust framework for identifying cell types, discovering rare cell populations, screening marker genes, exploring cellular developmental trajectories, and analysing cellular functions. ScRNA-Seq has also been extensively applied in animal science, particularly in studies involving domestic species of high economic importance. Using scRNA-Seq, animal researchers have investigated topics such as nutritional regulation, metabolic mechanisms, spermatogenesis, embryonic development, genetic breeding, and disease mechanisms in livestock. Through the construction of single-cell atlases for different animal organs, researchers have revealed the intricate cellular heterogeneity and gene expression variability present at the individual, organ, and tissue levels. This approach has facilitated deeper mechanistic investigations at the molecular level, shedding light on the underlying genetic basis of important traits and disease pathogenesis. Consequently, scRNA-Seq has made substantial and significant contributions to advancing our understanding of animal biology and is ultimately poised to enhance the quality of livestock products to meet increasing consumer demands. When analysing single-cell transcriptomic data, the optimal sample size should be determined based on the biological variation, statistical significance, cost and complexity of the data analysis. Accurate cell clustering and cell type annotation should consider background knowledge, as well as appropriate and stable algorithms. Currently, scRNA-Seq still faces several great challenges, ranging from sample processing to data analysis, and the integration of novel experimental methodologies and sequencing technologies is needed to probe and elucidate the intricate regulatory networks and causal relationships among diverse biomolecules in a multidimensional manner. Through continued innovation and interdisciplinary collaboration, single-cell transcriptomics holds the promise of unlocking new frontiers in animal research and fostering sustainable advancements in agricultural productivity and animal health.

### Supplementary Information

**Additional file 1**. Applications of single-cell transcriptomics in animal science.

## Data Availability

Not applicable.

## Abbreviations

Cg-like: Channel-gap-like
EndMT: Endothelial-to-mesenchymal transition
GIT: Gastrointestinal tract
PBMCs: Peripheral blood mononuclear cells
RNA-Seq: RNA-Sequencing
SCFA: Short-chain fatty acid
scRNA-Seq: Single-cell RNA sequencing
